# Serological and molecular surveillance of infectious bovine rhinotracheitis in Kazakhstan

**DOI:** 10.3389/fvets.2025.1734128

**Published:** 2026-01-06

**Authors:** Aiken Karabassova, Vladimir Kirpichenko, Raikhan Nissanova, Marat Turkeev, Akmerey Akylbay, Perizat Akshalova, Elvira Bashenova, Bakhyt Tulepov, Saltanat Mamanova, Saira Kaimoldina, Arailym Zhapbar, Fariza Ikramkulova, Aigul Kassen, Aisha Zharmukhametova, Zhandos Abay, Malik Yussupov, Kunsulu Zakarya, Aralbek Rsaliyev, Yergali Abduraimov, Zhibek Zhetpisbay, Galiya Sairambekova, Ainur Nurpeisova, Markhabat Kassenov

**Affiliations:** 1 Kazakh Scientific Research Veterinary Institute, LLP (KazSRVI), Almaty, Kazakhstan; 2 National Holding Qazbiopharm, JSC, Astana, Kazakhstan; 3 Department of Computer Science, Al-Farabi Kazakh National University, Almaty, Kazakhstan; 4 Masgut Aikimbayev National Scientific Center for Especially Dangerous Infections, Almaty, Kazakhstan

**Keywords:** BoHV-1, ELISA, infectious bovine rhinotracheitis, Kazakhstan, PCR, reference serum panels, surveillance

## Abstract

**Introduction:**

Infectious bovine rhinotracheitis (IBR), caused by bovine herpesvirus type 1 (BoHV-1), remains endemic in Kazakhstan. Despite the absence of vaccination, comprehensive multi-year data on the virus circulation have been limited.

**Methods:**

A three-year surveillance between 2023 and 2025 was conducted in unvaccinated cattle from all 17 administrative regions a total of 8,590 serum samples and 4,795 nasal swabs. Serological monitoring was performed using two validated ELISA systems (IDEXX IBR gE Ab Test and ID Screen® IBR Indirect ELISA). Molecular detection employed real-time PCR on nasal swabs, and virus-isolation attempts were carried out on Vero cell monolayers. National reference serum panels were developed and externally validated at the Friedrich-Loeffler-Institut (Germany).

**Results:**

Serological testing revealed consistently high antibody prevalence—69.13% (2023), 80.64% (2024), and 82.79% (2025)—with marked regional variation. PCR positivity was 11.2% (280/2,500) in 2024 but only 0.43% (10/2,295) in 2025, indicating subclinical circulation and intermittent viral shedding. All virus-isolation attempts were negative, consistent with low viral loads and latent infection. The validated serum panels achieved > 95% concordance with international reference sera and were successfully implemented for national QA/QC.

**Conclusion:**

These findings confirm persistent BoHV-1 endemicity in Kazakhstan and highlight the diagnostic and epidemiological framework necessary for harmonized surveillance, DIVA-compatible vaccination (Differentiating Infected from Vaccinated Animals), and progressive alignment with WOAH eradication programs.

## 1 Introduction

*Bovine herpesvirus type 1* (BoHV-1), the etiological agent of infectious bovine rhinotracheitis (IBR) and infectious pustular vulvovaginitis (IPV), remains a pathogen of considerable veterinary and economic concern worldwide. The virus, belonging to the genus Var*icellovirus* within the subfamily *Alphaherpesvirinae* (family *Herpesviridae*), causes respiratory, reproductive, and neonatal disease in cattle, resulting in significant production and trade losses (1). Its ability to establish latency in sensory neurons with reactivation and viral shedding under stress complicates eradication strategies (2, 3). Recent epidemiological studies highlight the persistence of BoHV-1 in multi-source herds and the role of animal movement, herd size, and replacement policies as major risk factors for introduction and maintenance of infection (4).

Despite the success of eradication programs in several European countries through test-and-cull and marker-vaccination (DIVA) strategies, BoHV-1 remains endemic in high-density livestock systems where biosecurity measures are inconsistently applied (5). The WOAH (World Organisation for Animal Health) Terrestrial Manual (Manual of Diagnostic Tests and Vaccines for Terrestrial Animals) (6) and recent program evaluations emphasize the essential role of harmonized diagnostic networks and laboratory quality-assurance frameworks that integrate serology, molecular detection, and national reference standards.

The diagnostic complexity of BoHV-1 stems from the narrow window of productive viral shedding, the prevalence of latent infections, and frequent failure to recover viable virus from field samples. These biological constraints necessitate molecular and serological surveillance as complementary pillars of epidemiological monitoring (7). Recently, novel approaches such as time-resolved fluorescence lateral flow immunochromatographic strips have demonstrated high concordance with real-time PCR for BoHV-1 detection, providing practical solutions for field settings (8). Molecular insights into the genetic diversity of circulating strains—supported by whole-genome sequencing and phylogenetic analyses—underscore the ongoing evolution and geographic structuring of BoHV-1 (9).

Marker (gE-deleted) ELISAs remain indispensable for differentiating infected from vaccinated animals, forming the backbone of DIVA-based programs (10). Comparative vaccine trials have confirmed the safety and immunogenicity of BoHV-1 marker vaccines, including combinations with respiratory pathogens (11) and alternative host studies in water buffaloes (12). These data reinforce that even under vaccination, seromonitoring must remain continuous, using validated reagents and harmonized interpretation criteria.

In Kazakhstan (2021–2022 and in the first half of 2023), BoHV-1 continues to circulate widely, with repeated notifications to WOAH and confirmed seropositivity in both commercial and smallholder herds. National data indicate long-term endemicity, limited virus isolation success, and a lack of unified QA/QC materials for inter-laboratory comparison (13). Within the framework of the Scientific and Technical Program (STP) IRN BR218004/0223 “Improvement of biological safety measures in Kazakhstan: counteraction to dangerous and especially dangerous infections” for the years 2023–2025, a comprehensive multi-year study was conducted to assess the epizootic status of infectious bovine rhinotracheitis (IBR) under field conditions across all administrative regions of Kazakhstan, with a focus on non-vaccinated cattle populations.

The objectives of this study were to:

Conduct three-year serological surveillance (2023–2025) across 17 regions of Kazakhstan using validated ELISA systems;
Perform molecular (rRT-PCR) monitoring on paired nasal swabs to detect active viral circulation;
Attempt virus isolation on Vero cell cultures to evaluate recovery feasibility from field material;
Develop and externally validate national reference serum panels (positive and negative) at the Friedrich-Loeffler-Institut (FLI, Germany), confirming equivalence to international reference sera and enabling their use in field seromonitoring for QA/QC purposes.

This study integrates serological, molecular, and virological methods with validated quality-assurance materials, offering a high-resolution depiction of BoHV-1 epidemiology in Kazakhstan. By aligning with current international standards and methodological innovations (14), it contributes to strengthening the national surveillance infrastructure and advancing Kazakhstan’s long-term goal of establishing a WOAH-aligned reference system for IBR control and eradication.

## 2 Materials and methods

### 2.1 Study design and sampling strategy

Between 2023 and 2025, a nationwide multi-year surveillance program for Bovine herpesvirus 1 (BoHV-1) was conducted across all 17 administrative regions of the Republic of Kazakhstan, encompassing both commercial and smallholder cattle herds. The program incorporated retrospective 2023 data sourced from national monitoring reports, as well as original 2024–2025 data generated through active field sampling and laboratory testing conducted at KazSRVI. The study was implemented under the framework of the Scientific and Technical Program (STP) IRN BR218004/0223 “Improvement of biological safety measures in Kazakhstan: counteraction to dangerous and especially dangerous infections” (2023–2025). Sampling was designed to capture broad regional and production-type diversity. Convenience sampling was used, prioritizing herds with available epidemiological histories and voluntary participation. Importantly, all animals included in this study were non-vaccinated against BoHV-1. Nasal swabs were collected from the same animals as the blood samples, ensuring paired serological and virological assessment. All samples were transported at +4 °C and processed within 48 h at the Virology Laboratory, Kazakh Scientific Research Veterinary Institute (KazSRVI, Almaty).

### 2.2 Serological testing

Serological screening for antibodies against BoHV-1 was performed using two commercial enzyme-linked immunosorbent assay (ELISA) kits:

IDEXX IBR gE Ab Test (IDEXX Laboratories, Liebefeld-Bern, Switzerland) – a blocking ELISA targeting the gE glycoprotein to differentiate infected from vaccinated animals (DIVA principle).

ID Screen® IBR Indirect ELISA (ID. Vet, Grabels, France) – for broad serological detection of anti-BoHV-1 antibodies.

All procedures were performed in accordance with the manufacturers’ instructions. Serum optical densities (OD) were measured at 450 nm using a Thermo Scientific Multiskan™ FC reader. Samples were considered positive when the sample-to-negative control ratio (S/N %) or sample-to-positive control ratio (S/P %) fell within the thresholds recommended by the manufacturer. Internal quality control included duplicate testing of 10% of samples, and results were validated using reference positive and negative sera developed at KazSRVI (see Section 2.5).

### 2.3 Molecular detection (rRT-PCR)

Molecular screening was conducted using real-time PCR to detect BoHV-1 DNA in nasal swabs. Two validated assays were applied in parallel for quality comparison:

VetMAX™ IBR/BHV-1 Reagents (Thermo Fisher Scientific, USA), PCR-RHINOTRACHEITIS-CATTLE FACTOR Bovine herpes virus 1, BoHV-1 (Russia).

Nucleic acids were extracted using the QIAamp Viral RNA Mini Kit (Qiagen, Germany) following manufacturer instructions. PCR reactions were carried out on a QuantStudio™ 5 Real-Time PCR System (Applied Biosystems). Amplification conditions were 95 °C for 10 min, followed by 40 cycles of 95 °C for 15 s and 60 °C for 1 min.

Samples were classified as positive when cycle threshold (Ct) ≤ 38, equivocal for 38 < Ct ≤ 40, and negative when no amplification occurred. Positive and negative extraction controls were included in every run.

### 2.4 Virus isolation attempts

Attempts to isolate BoHV-1 from rRT-PCR–positive nasal swabs were conducted using Vero cell cultures (ATCC CCL-81). Swab supernatants were clarified by centrifugation at 3,000 × g for 10 min, filtered through 0.45 μm syringe filters, and inoculated onto confluent Vero monolayers in 25 cm^2^ flasks. Cultures were incubated at 37 ± 1 °C with 5% CO₂ and monitored for cytopathic effect (CPE) for up to 7 days. Each sample underwent three consecutive blind passages. A reference BoHV-1 strain (Cooper-1, ATCC VR-864) was used as a positive control. No CPE was observed in any of the field isolates, and all cultures remained PCR-negative, confirming the absence of replicating virus in tested materials.

### 2.5 Development and validation of national reference serum panels

To support assay harmonization and QA/QC, positive and negative reference serum panels for IBR were developed at KazSRVI in 2024. Positive sera were obtained from naturally infected, seropositive cattle confirmed by both indirect and blocking ELISAs and further verified by PCR. Negative sera were collected from confirmed BoHV-1–free animals originating from herds with no IBR history and consistent ELISA-negative results. All sera were heat-inactivated (56 °C, 30 min), pooled into three titer groups (strong positive, weak positive, and negative), aliquoted, and stored at −20 °C. The panels were externally validated at the Friedrich-Loeffler-Institut (FLI, Germany) against international reference sera provided by WOAH-recognized laboratories. Validation confirmed their equivalence in terms of signal intensity and diagnostic concordance (> 95%) across tested ELISA platforms. The panels were subsequently implemented during 2025 seromonitoring to verify routine diagnostic performance in Kazakh laboratories.

### 2.6 Statistical analysis

Data were analyzed using GraphPad Prism v9.5.1 (GraphPad Software, USA) and R v4.3.1 (R Foundation for Statistical Computing, Austria). Seroprevalence and PCR positivity rates were calculated with 95% confidence intervals (CI) using the Wilson method. Regional differences in prevalence were assessed using Pearson’s chi-square test or Fisher’s exact test where applicable. The *Z*-test for two proportions was applied to evaluate annual variation between surveillance years. A *p*-value < 0.05 was considered statistically significant.

## 3 Results

### 3.1 Overview of the three-year surveillance (2023–2025)

A comprehensive serological and molecular surveillance of infectious bovine rhinotracheitis (IBR) was conducted across all 17 administrative regions of Kazakhstan during 2023–2025. The program included retrospective data for 2023 derived from national monitoring reports and original data for 2024–2025 obtained through active field sampling and laboratory testing performed at KazSRVI. Each year included sampling of unvaccinated cattle herds representing both commercial and smallholder production systems.

**Figure 1:**
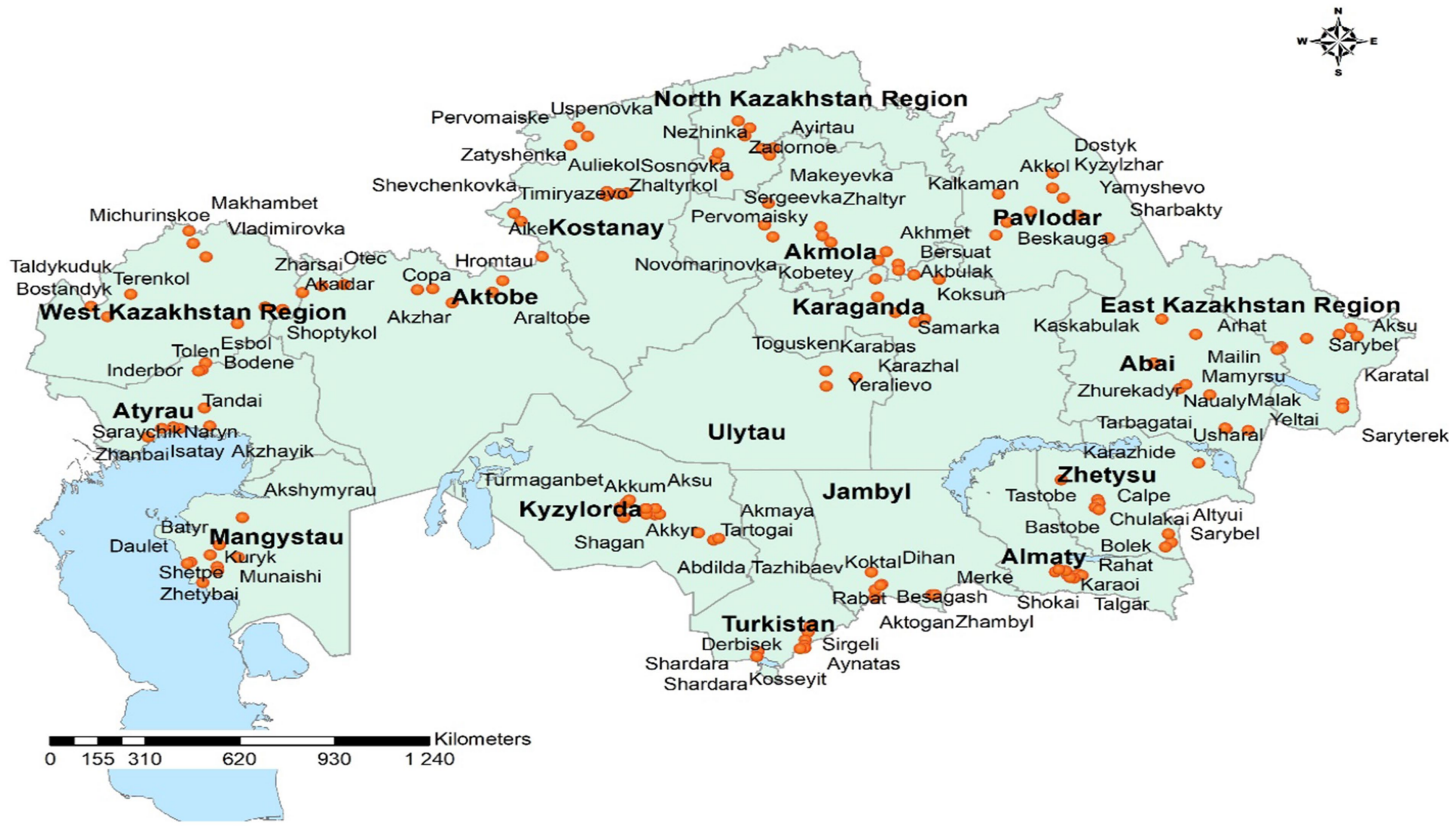
Geographic distribution of IBR (BoHV-1) sampling sites across Kazakhstan, 2024.

**Figure 2:**
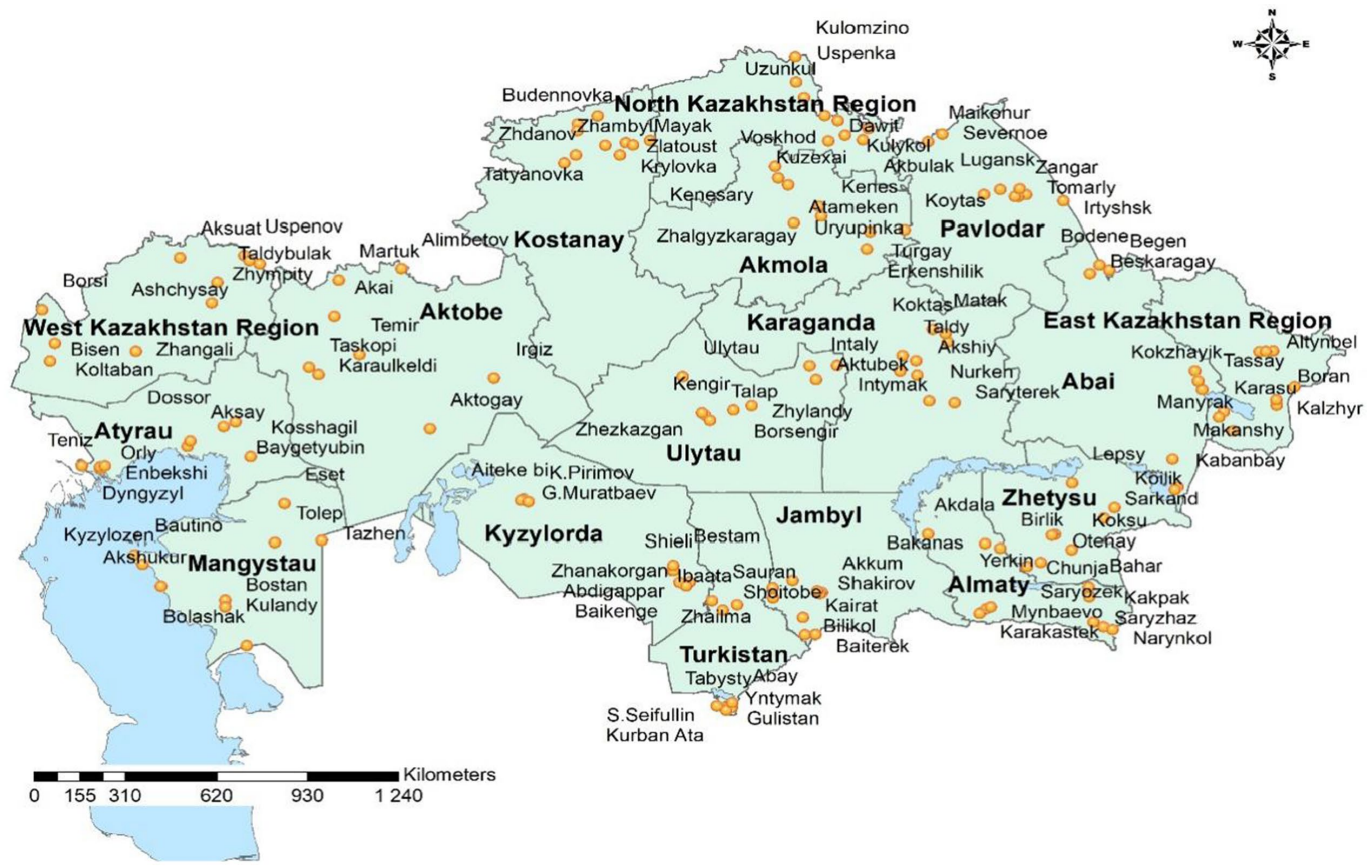
Geographic distribution of IBR (BoHV-1) sampling sites across Kazakhstan, 2025.

Overall, a total of 8,590 serum samples and 4,795 nasal swabs from cattle aged 1 to 10 years were analyzed over the three-year period from 306 epidemiological units and 112 districts (Figures 1, 2). The combined dataset allowed for comparative assessment of antibody prevalence, viral nucleic acid detection, and laboratory validation of diagnostic reagents.

### 3.2 Serological surveillance (2023–2025)

A total of 3,795 serum samples collected in 2023, 2,500 in 2024, and 2,295 in 2025 were examined using commercial ELISA kits (IDEXX IBR gE Ab Test and ID Screen® IBR Indirect ELISA). The results demonstrated persistent circulation of IBR virus throughout the national herd.

According to national surveillance data from the Ministry of Agriculture, the mean annual seroprevalence was 69.13% in 2023 across 14 regions, whereas our own field investigations estimated 80.64% in 2024 and 82.79% in 2025 (Table 1). This indicates a moderate increase in infection prevalence from 2023 to 2025, while maintaining consistently high levels of infection across the country. The observed variability is likely due to regional differences in management practices, herd size, and animal movement patterns rather than abrupt epidemiological changes.

**Table 1:** Summary of serological results by year and region (2024–2025).

Region	2024 year				2025 year			
	Seroprev. (%)	95% CI (Wilson)	*p*-value (Binom.)	*p*-value (Fisher)	Seroprev. (%)	95% CI (Wilson)	*p*-value (Binom.)	*p*-value (Fisher)
Almaty	55.55	[47.14, 63.67]	1.0000	1.0000	55.83	[46.90, 64.40]	1.0000	1.0000
Zhetysu	100	[97.23, 100]	0.0000	0.0000	65.18	[56.83, 72.70]	1.0000	1.0000
Zhambyl	90.37	[84.22, 94.29]	0.0016	0.0013	100	[97.23, 100]	0.0000	0.0000
Turkestan	91.11	[85.11, 94.84]	0.0007	0.0005	67.41	[59.11, 74.74]	0.9999	1.0000
Kyzylorda	39.44	[32.60, 46.73]	1.0000	1.0000	78.51	[70.85, 84.61]	0.9202	0.9264
Aktobe	87.41	[80.76, 91.99]	0.0256	0.0226	90.37	[84.22, 94.29]	0.0095	0.0080
Mangystau	95.55	[90.64, 97.95]	0.0000	0.0000	81.48	[74.09, 87.13]	0.7035	0.7083
West Kazakhstan	94.81	[89.68, 97.47]	0.0000	0.0000	96.29	[91.62, 98.41]	0.0000	0.0000
Atyrau	100	[97.23, 100]	0.0000	0.0000	85.92	[79.06, 90.80]	0.1991	0.1914
Akmola	89.62	[83.34, 93.72]	0.0035	0.0029	88.14	[81.61, 92.57]	0.0575	0.0522
North Kazakhstan	65.92	[57.59, 73.38]	0.1000	1.0000	98.51	[94.76, 99.59]	0.0000	0.0000
Kostanay	67.41	[59.11, 74.74]	0.9999	0.9999	96.00	[91.55, 98.15]	0.0000	0.0000
Pavlodar	94.81	[89.68, 97.47]	0.0000	0.0000	69.62	[61.42, 76.75]	0.9999	1.0000
Karaganda	83.33	[78.10, 87.52]	0.1650	0.1525	89.62	[83.34, 93.72]	0.0186	0.0160
Ulytau	55.26	[48.16, 62.16]	1.0000	1.0000	80.00	[72.46, 85.88]	0.8351	0.8421
East Kazakhstan	94.81	[89.68, 97.47]	0.0000	0.0000	71.11	[62.97, 78.09]	0.9997	0.9998
Abai	87.41	[80.76, 91.99]	0.025579	0.0226	88.88	[82.48, 93.15]	0.0338	0.0299
Total	80.64	[79.04, 82.14]	-	-	82.79	[81.19, 84.28]	**-**	**-**

**Figure 3:**
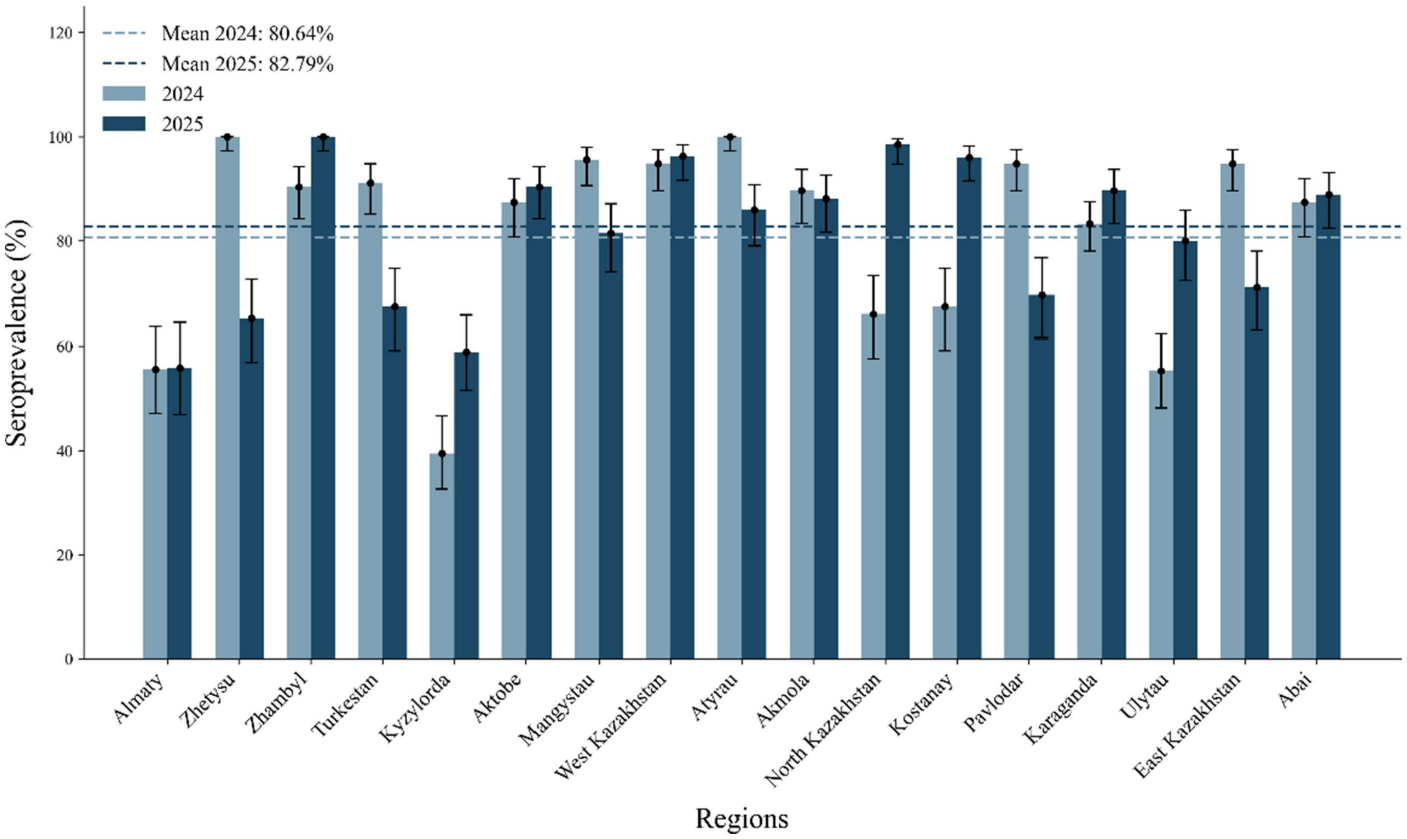
Regional distribution of IBR seroprevalence, 2024–2025.

Marked spatial heterogeneity was recorded across the regions. In 2024, seroprevalence ranged from 39.44% (Kyzylorda) to 100% (Atyrau, Zhetysu). Similar patterns persisted in 2025, with high seropositivity retained in southern and western regions (Figure 3).

Diagnostic concordance between the two ELISA systems remained high across all years (*κ* = 0.93, 95% CI: 0.90–0.96), confirming analytical reliability. Retesting of 10% of samples yielded identical qualitative outcomes, indicating high reproducibility.

### 3.3 Molecular detection by rRT-PCR (2024–2025)

Real-time PCR testing of nasal swabs was introduced in 2024 to detect circulating BoHV-1 DNA. In 2024, 2,500 swabs were analyzed, with 280 testing positive (11.2%). In 2025, 2,295 swabs were examined, yielding a comparable proportion of positive results 10 (0.43%).

**Figure 4:**
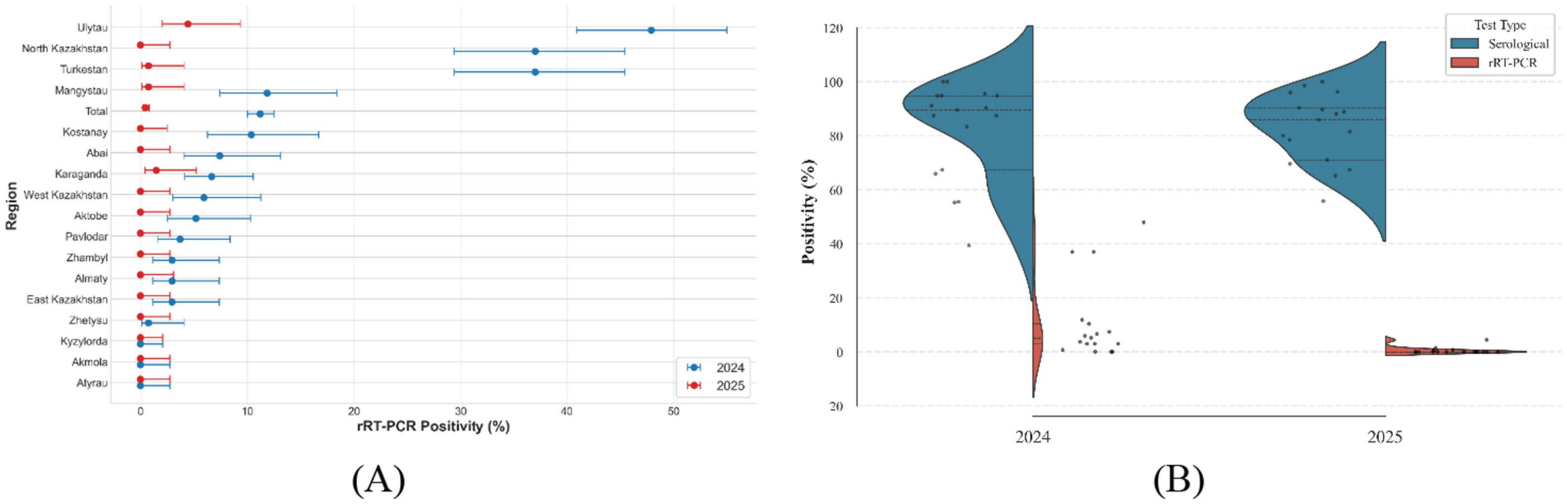
Distribution of rRT-PCR positivity **(A)** by region; **(B)** comparison with seroprevalence.

Ct values for positive samples ranged from 23.6 to 37.8 (median 31.2), consistent with subclinical or latent infection status. Both the VetMAX™ IBR/BHV-1 Reagents (Thermo Fisher Scientific, Waltham, USA) and the PCR-RHINOTRACHEITIS-CATTLE FACTOR (Russia) PCR kit demonstrated equivalent performance (*r* = 0.98, *p* < 0.001), confirming the reliability of the domestically developed assay (Figure 4; Table 2).

**Table 2:** Molecular detection of BoHV-1 by rRT-PCR, 2024–2025 (regional breakdown).

Region	2024 year				2025 year			
	Samples tested by PCR-RV (nasal swabs)	PCR-RV positive	Infection rate, %	95% CI (Wilson)	Samples tested by PCR-RV (nasal swabs)	PCR-RV positive	Infection rate, %	95% CI (Wilson)
Almaty	135	4	2.96	[1.16, 7.37]	120	0	0	[0.00, 3.10]
Zhetysu	135	1	0.74	[0.13, 4.08]	135	0	0	[0.00, 2.77]
Zhambyl	135	4	2.96	[1.16, 7.37]	135	0	0	[0.00, 2.77]
Turkestan	135	50	37.03	[29.36, 45.44]	135	1	0.74	[0.13, 4.08]
Kyzylorda	180	0	0	[0.00, 2.09]	180	0	0	[0.00, 2.09]
Aktobe	135	7	5.18	[2.53, 10.32]	135	0	0	[0.00, 2.77]
Mangystau	135	16	11.85	[7.43, 18.39]	135	1	0.74	[0.13, 4.08]
West Kazakhstan	135	8	5.92	[3.03, 11.26]	135	0	0	[0.00, 2.77]
Atyrau	135	0	0	[0.00, 2.77]	135	0	0	[0.00, 2.77]
Akmola	135	0	0	[0.00, 2.77]	135	0	0	[0.00, 2.77]
North Kazakhstan	135	50	37.03	[29.36, 45.44]	135	0	0	[0.00, 2.77]
Kostanay	135	14	10.37	[6.28, 16.66]	150	0	0	[0.00, 2.50]
Pavlodar	135	5	3.70	[1.59, 8.38]	135	0	0	[0.00, 2.77]
Karaganda	240	16	6.66	[4.14, 10.55]	135	2	1.48	[0.41, 5.24]
Ulytau	190	91	47.89	[40.90, 54.97]	135	6	4.44	[2.05, 9.36]
East Kazakhstan	135	4	2.96	[1.16, 7.37]	135	0	0	[0.00, 2.77]
Abai	135	10	7.40	[4.07, 13.10]	135	0	0	[0.00, 2.77]
Total	2,500	280	11.2	[10.02, 12.50]	2,295	10	0.43	[0.24, 0.80]

### 3.4 Virus isolation

Virus isolation attempts were conducted on all rRT-PCR–positive samples from 2024–2025 using Vero cell monolayers (ATCC CCL-81). Three serial blind passages were performed under standard incubation conditions (37 °C, 5% CO₂). No cytopathic effect (CPE) was observed in any field sample, and all cultures remained PCR-negative after the third passage. The reference strain Cooper-1 (ATCC VR-864) produced characteristic CPE within 48 h, confirming cell line susceptibility.

**Figure 5:**
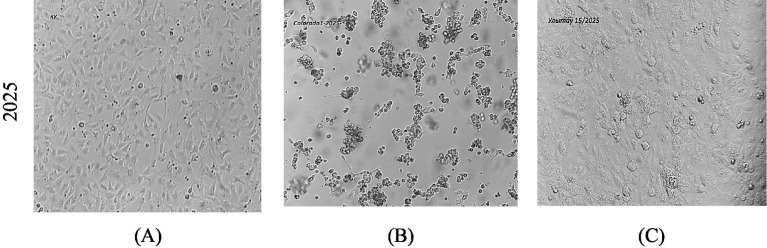
Representative micrographs **(A)** uninfected Vero cells; **(B)** Cooper-1 CPE; **(C)** field sample (no CPE).

The failure to isolate viable virus likely reflects low viral loads or sampling during non-excretion phases, consistent with the latent infection dynamics of BoHV-1 (Figure 5; Table 3).

**Table 3:** Summary of virus isolation attempts, 2024–2025.

Year	Region	Total number of samples	Cattle sera positive by IRT (PCR-RV)	Selected for virus isolation in cell culture (count/sample ID)
2024	Karaganda	240	16	4/(735, 732, 729, 578)
	West Kazakhstan	135	8	4/(397, 399, 434, 504)
	Almaty	135	4	2/(1,687, 1,690)
	Mangystau	135	16	7/(747, 749, 750, 759,817, 829, 831)
	Total	645	44	17
2025	Turkestan	135	1	1/10
	West Kazakhstan	135	1	1/12
	Karaganda	135	2	2/9, 11, 13
	Ulytau	135	6	6/2, 3, 4, 5, 9, 15

### 3.5 Validation of reference serum panels

**Figure 6:**
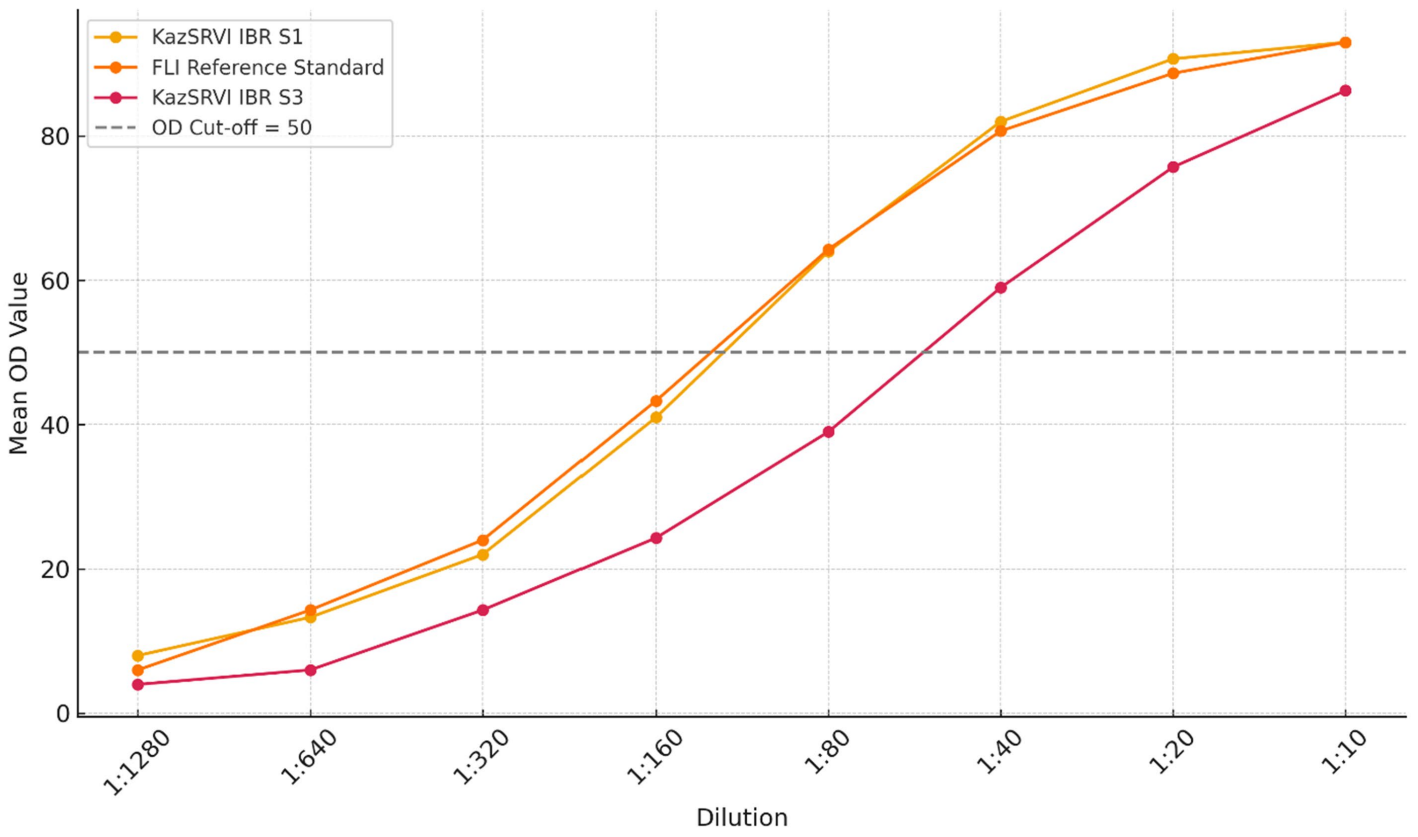
Comparative ELISA signal intensities: national vs. international reference sera (FLI validation).

In 2024, KazSRVI developed national reference serum panels comprising positive, weak-positive, and negative sera to support standardization of ELISA-based IBR diagnostics. These panels were externally validated at the Friedrich-Loeffler-Institut (FLI, Germany) against international reference sera recognized by WOAH. Validation confirmed diagnostic equivalence (>95% concordance) across multiple ELISA platforms. During field use in 2025, OD variation between replicates remained below 5% after 6 months of storage, confirming high stability (Figure 6; Table 4).

**Table 4:** Composition of national reference serum panels and validation outcomes.

Code/ID	Serum name	IBR Ab status (per FLI certificate)	Proposed role in the panel	Validation assays (FLI/IDEXX)
KazSRVI IBR 1	Abai	Strong positive	Reference “strong positive”	IDEXX IBR gB blocking ELISA; IDEXX IBR gE blocking ELISA; IDEXX whole virus IBR Trachitest ELISA
KazSRVI IBR 2	Kurban Ata	Strong positive	Reference “strong positive”	IDEXX IBR gB blocking ELISA; IDEXX IBR gE blocking ELISA; IDEXX whole virus IBR Trachitest ELISA
KazSRVI IBR 3	Mynbay	Positive	Reference “positive”	IDEXX IBR gB blocking ELISA; IDEXX IBR gE blocking ELISA; IDEXX whole virus IBR Trachitest ELISA
KazSRVI IBR 4	Seifullin	Strong positive	Reference “strong positive”	IDEXX IBR gB blocking ELISA; IDEXX IBR gE blocking ELISA; IDEXX whole virus IBR Trachitest ELISA
KazSRVI IBR 5	Boxmix-2	Strong positive	Reference “strong positive”	IDEXX IBR gB blocking ELISA; IDEXX IBR gE blocking ELISA; IDEXX whole virus IBR Trachitest ELISA

### 3.6 Summary of trends and observations

The three-year surveillance confirmed the endemic presence of BoHV-1 infection across Kazakhstan. The transition from 2023 to 2025 was characterized by an increase in seropositivity, suggesting active virus circulation in unvaccinated herds.

Molecular detection corroborated ongoing subclinical circulation of BoHV-1, whereas the absence of viable virus in culture underscored the predominance of latent infections during the study period. The validation of domestic reference serum panels provides an important step toward national assay harmonization and alignment with international standards.

## 4 Discussion

This study was designed to provide a three-year, nationwide assessment of bovine herpesvirus type 1 (BoHV-1) circulation in unvaccinated cattle herds, combining serological and molecular surveillance across all 17 regions of Kazakhstan. Serological monitoring demonstrated a high and persistent level of exposure, with seroprevalence reaching 69.13% in 2023, 80.64% in 2024 (2,016/2,500), and 82.79% in 2025 (1,662/2,295). Data for 2023 were generated using the same commercial ELISA platforms, ensuring methodological comparability. This trend—an increase from baseline to 2024 followed by a modest decline in 2025—reflects continuous virus circulation and possible herd-immunity saturation. Spatial heterogeneity was pronounced: Atyrau and Zhetysu achieved 100% seropositivity, whereas Kyzylorda remained lowest (39.44%), underscoring regional differences in management practices, biosecurity, and livestock movement networks. Similar geographic heterogeneity has been described in European control programs, where herd size and purchase frequency represent major risk factors for persistence (14). Spatial heterogeneity likely reflects regional variation in herd density, production structure, and biosecurity capacity, as supported by previous epidemiological studies in Kazakhstan and comparable livestock systems in Central Asia. The sustained high prevalence into 2025 suggests entrenched endemicity and supports the need for region-specific control strategies rather than uniform national measures.

Parallel molecular surveillance in 2024–2025 provided complementary evidence of infection dynamics. In 2024, 11.2% of nasal swabs (280/2,500) tested positive by real-time PCR, with Ct values from 23.6 to 37.8 (median ≈ 31.2), consistent with low-level mucosal replication during subclinical or reactivation phases. In 2025, only 10 of 2,295 swabs (≈ 0.43%) were positive despite equivalent geographic coverage. This decline may reflect the episodic nature of viral shedding and possibly differences in sampling windows or temporal viral load among herds, rather than assay failure. The excellent correlation between the VetMAX™ IBR/BHV-1 Reagents and the The excellent correlation between the VetMAX™ IBR/BHV-1 Reagents and the PCR-RHINOTRACHEITIS-CATTLE FACTOR assay (*r* = 0.98, *p* < 0.001) confirms analytical robustness and supports confidence in these field results. Assay (*r* = 0.98, *p* < 0.001) confirms analytical robustness and supports confidence in these field results. Comparable year-to-year fluctuations in BoHV-1 detection have been reported in longitudinal studies of subclinical herds (15).

Convenience sampling represents an important limitation of this study. Voluntary herd participation and the availability of epidemiological records may have resulted in uneven regional and production-type coverage, potentially affecting estimates of true prevalence. Although the sampling included all administrative regions and both commercial and smallholder herds, the data may not fully represent the national cattle population.

Attempts to isolate virus from PCR-positive material yielded no recoverable field strains despite three blind passages in Vero cells and clear cytopathic response of the reference Cooper-1 strain, confirming culture competency. The inability to obtain isolates likely reflects low viral titers or sampling outside active shedding phases, consistent with the latent-infection biology of alphaherpesviruses. According to the WOAH Terrestrial Manual (6), real-time PCR is the primary method for diagnosis and surveillance of IBR, while virus isolation is inherently insensitive under field conditions. Therefore, the reliance on molecular tools rather than viropathic recovery is both justified and methodologically aligned with current global practice (16).

A key output of this work was the development and external validation of national reference serum panels at the Friedrich-Loeffler-Institut (FLI, Germany). Demonstrating >95% diagnostic concordance with international reference sera across multiple ELISA platforms, these panels substantially strengthen Kazakhstan’s diagnostic QA/QC system. Their successful field deployment in 2025 supports sustainable assay harmonization and inter-laboratory comparability—core performance metrics in modern surveillance evaluation frameworks (14). This achievement represents an essential infrastructural milestone for future accreditation and for alignment with international reporting standards.

The strengths of the present study include its broad temporal and geographic scope, use of validated commercial assays with proven reproducibility, and transparent reporting of negative virus-isolation outcomes. Collectively, these features provide one of the most comprehensive datasets on BoHV-1 epidemiology in Central Asia. However, several limitations should be acknowledged. The use of convenience sampling and non-paired sera/swab design limits individual-level inference and likely contributes to observed serology–PCR discordance. Differences in sampling season and herd composition across years may have influenced PCR yield, while the 2023 dataset—although methodologically consistent—originated from national monitoring records and may differ subtly in field logistics. Finally, the absence of full-genome sequencing constrains interpretation of strain diversity and introduction routes, though partial gC sequencing established technical feasibility for future genomic work (17). These limitations highlight the need for longitudinal paired sampling and harmonized protocols in forthcoming surveillance cycles (18–20).

The observed decoupling between high seroprevalence and markedly lower PCR positivity in 2025 emphasizes that BoHV-1 infection in Kazakhstan is entering a mature endemic phase, dominated by latent carriers with sporadic reactivation. Future surveillance should therefore incorporate serial swabbing within sentinel herds, apply output-based freedom-from-infection metrics at regional scale (7), and evaluate DIVA-compatible vaccination strategies targeted to high-risk areas (11). Integration of whole-genome sequencing will enable tracing of transmission clusters and comparison with global BoHV-1 lineages, advancing molecular epidemiology and risk modeling (17). Altogether, this study provides a robust diagnostic and analytical foundation for evidence-based control of BoHV-1 in Kazakhstan, fostering harmonized surveillance, regional risk mapping, and progressive alignment with WOAH-recommended eradication pathways.

## Data Availability

The original contributions presented in the study are included in the article/supplementary material, further inquiries can be directed to the corresponding author.
